# The Versatility of Serine Proteases from Brazilian *Bothrops* Venom: Their Roles in Snakebites and Drug Discovery

**DOI:** 10.3390/biom15020154

**Published:** 2025-01-21

**Authors:** Marcela Romanazzi, Eloise T. M. Filardi, Geovanna M. M. Pires, Marcos F. Cerveja, Guilherme Melo-dos-Santos, Isadora S. Oliveira, Isabela G. Ferreira, Felipe A. Cerni, Norival Alves Santos-Filho, Wuelton M. Monteiro, José R. Almeida, Sakthivel Vaiyapuri, Manuela B. Pucca

**Affiliations:** 1 Graduate Program in Bioscience and Biotechnology Applied to Pharmacy, School of Pharmaceutical Sciences, São Paulo State University (UNESP), Araraquara 19060-900, Brazil; marcela.romanazzi@unesp.br (M.R.); marcos.cerveja@unesp.br (M.F.C.);; 2 Department of Clinical Analysis, School of Pharmaceutical Sciences, São Paulo State University (UNESP), Araraquara 19060-900, Brazil; 3 Department of Biomolecular Sciences, School of Pharmaceutical Sciences of Ribeirão Preto, University of São Paulo, Ribeirão Preto 19040-903, Brazil; 4 Medical School, Federal University of Roraima, Boa Vista 69310-000, Brazil; 5 Department of Biochemistry and Organic Chemistry, Institute of Chemistry, São Paulo State University (UNESP), Araraquara 14800-060, Brazil; 6 Department of Teaching and Research, Dr. Heitor Vieira Dourado Tropical Medicine Foundation, Manaus 69850-000, Brazil; wueltonmm@gmail.com; 7 School of Pharmacy, University of Reading, Reading RG6 6UB, UK

**Keywords:** serine protease, coagulation, snakebite, venom

## Abstract

Serine proteases are multifunctional and versatile venom components found in viper snakes, including the *Bothrops* species, a widely distributed genus notorious for causing the highest number of snakebites across Latin America. These enzymes, representing a significant fraction of *Bothrops* venom proteomes, exhibit a wide range of biological activities that influence blood coagulation, fibrinolysis, and inflammation. This review provides a comprehensive overview of serine proteases, with a particular focus on those found in the venom of Brazilian *Bothrops* snakes. The discussion begins with a summary of snake species found in Brazil and their medical relevance. Specifically addressing the *Bothrops* genus, this review explores the distribution of these species across Brazilian territory and their associated medical importance. Subsequently, the article investigates the biochemistry of *Bothrops* venoms and the clinical manifestations induced by envenomation. Finally, it offers an in-depth discussion on the serine proteases, highlighting their biochemical properties, mechanisms of action, and potential therapeutic applications. Furthermore, this review provides an in-depth exploration of the diverse serine proteases found in *Bothrops* venoms and their functional significance, from thrombin-like effects to potent fibrinogenolytic actions, which determine the clinical manifestations of envenomation. This review delves into the evolutionary adaptations and biochemical diversity of serine proteases in *Bothrops* venoms, emphasizing their critical roles in venom functionality and the resulting pathophysiological effects. Additionally, it opens new avenues for utilizing these enzymes in biomedical applications, underscoring their potential beyond toxinology.

## 1. Introduction

Earth is home to a vast array of animal species, many of which remain undiscovered. Among the identified species, approximately 15% (~220,000) of the planet’s total animal diversity possess venom [1]. These bioactive cocktails, mainly composed of peptides and proteins, are essential for the survival and predatory strategies of many animals. Snakes stand out as some of the most studied venomous creatures in the world [2]. The intrigue and fear surrounding snakes date back to ancient times, with one of the earliest documented treatises on snakebite envenomation found in the Brooklyn Medical Papyrus of ancient Egypt [3].

Snake venom has facilitated the evolutionary shift from mechanical prey capture to a more sophisticated and efficient biomolecule-based strategy [4]. Regarded as natural libraries of bioactive compounds, still underexplored in many aspects, venoms hold a vast array of potential therapeutic applications [5,6]. From a biomolecular point of view, they consist of a mixture of components ranging from 20 to over 100, with the majority (>90%) being peptides and proteins. These compounds may exhibit neurotoxic, hemotoxic, and cytotoxic properties as dominant biological activities, depending on the snake species [7,8].

Several peptides and proteins isolated from snake venoms exhibit multiple therapeutic applications [9]. Some of them act as thrombolytic agents, antimicrobials, and antiviral compounds, being effective against different viruses such as herpes simplex, dengue, and yellow fever. Antiparasitic and antifungal properties have also been reported for venoms [10]. In this way, snake venom can be seen as a complex reservoir of medicinal substances. However, it is important to note that less than 0.01% of these proteins have been properly identified and characterized [11].

In *Bothrops* snake species, phospholipase A_2_ (PLA_2_), metalloproteases (SVMPs), and serine proteases (SVSPs) are dominant, comprising approximately 70% of the total proteins of snake venom [7,8]. SVSPs comprise a superfamily of proteolytic enzymes with multifunctional activities, being widely found in eukaryotes, prokaryotes, archaea, and viruses [12]. They can be found in the venoms of many snake families, including Viperidae, Crotalidae, Elapidae, and Colubridae [13,14]. However, the occurrence of serine proteases in elapid venoms is rare, and its relative abundance is very low. On the other hand, SVSPs are abundant in viper venoms, with a multiplicity of isoforms exhibiting different primary structures and biological actions [15,16]. Evolutionary studies have demonstrated that serine proteases may have undergone gene divergence and duplication, leading to alterations in their biological properties, thereby enabling the acquisition of their multiple functions [13].

SVSPs have a proteolytic nature, significantly impacting the breakdown of biologically active proteins, primarily those involved in hemostatic disturbances observed during snake envenomation. In this context, this review presents insights into *Bothrops* snakes worldwide, with a focus on the venom compositions of Brazilian snakes. Of particular interest, we review an expanding body of the literature describing the biochemical features and biological properties of SVSPs. We discuss how an in-depth investigation of these proteolytic enzymes can enhance our understanding of snakebite pathology and its treatment. Finally, we explore innovative avenues for translating SVSPs into drug discovery and diagnostic approaches.

## 2. Brazilian Snakes: An Overview of Species, Medical Significance, and Envenomation

**Figure 1:**
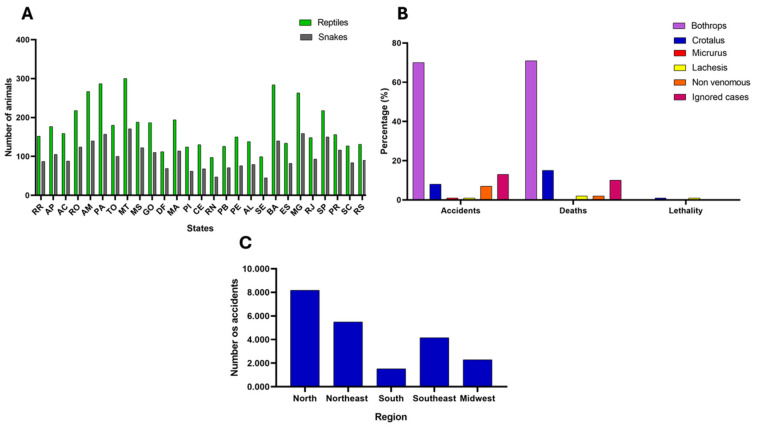
A geographical snapshot of Brazil’s reptile diversity and epidemiology of snakebites. (**A**) Number of reptiles versus snakes across states in Brazil (2022). RR—Roraima, AP—Amapá, AC—Acre, RO—Rondônia, AM—Amazonas, PA—Pará, TO—Tocantins, MT—Mato Grosso, MS—Mato Grosso do Sul, GO—Goiás, DF—Distrito Federal, MA—Maranhão, PI—Piauí, CE—Ceará, RN—Rio Grande do Norte, PB—Paraíba, PE—Pernambuco, AL—Alagoas, SE—Sergipe, BA—Bahia, ES—Espirito Santo, MG—Minas Gerais, RJ—Rio de Janeiro, SP—São Paulo, PR—Paraná, SC—Santa Catarina, and RS—Rio Grande do Sul. Adapted from [19]. (**B**) Snakebites in Brazil according to snake genera (2022). Bothrops: responsible for 69.61% of accidents, 71.28% of deaths, and a lethality rate of 0.33%. Crotalus: responsible for 7.88% of accidents, 14.89% of deaths, and a lethality rate of 0.6%. Micrurus: responsible for 1.15% of accidents, 0% of deaths, and a 0% lethality rate. Lachesis responsible for 0.99% of accidents, 2% of deaths, and a 2.13% lethality rate. Other snakes are responsible for 7.32% of accidents, 9.57% of deaths, and a 0.09% lethality rate. A total of 13.06% of accidents were not reported, along with 9.57% of deaths and a 0.23% lethality rate. Note that the number of deaths is related to the number of accidents from each snake genus, and the lethality rate represents the proportion of deaths attributed to a specific snake genus relative to all recorded snakebite incidents. (**C**) Bothropic snakebites of Brazil by region (2023). Data obtained from [20].

The Brazilian biomes are home to a significant portion of the world’s biodiversity. They boast high levels of species richness and endemism, making them critical biodiversity hotspots [17]. Currently, more than 10,700 species of reptiles are recognized worldwide, with Brazil accounting for 4746 reptile species according to the data from the Brazilian Society of Herpetology (Figure 1A), with 40% of the recorded snake species being endemic to the country (a total of 179, including species and subspecies) [18].

**Figure 2:**
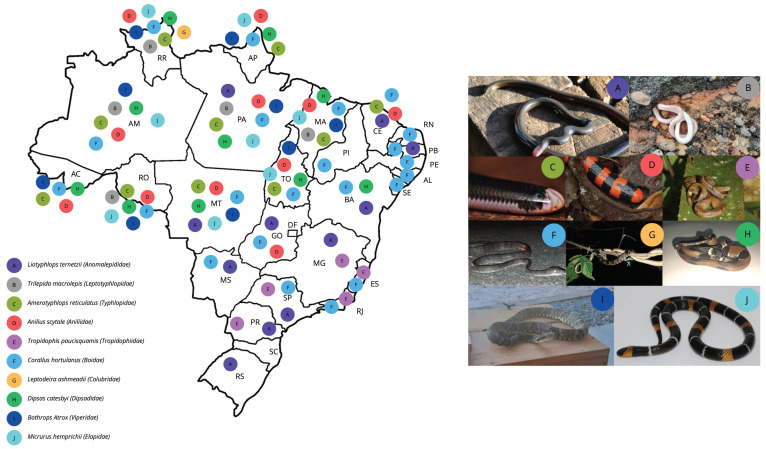
Examples of diversity of snake families in Brazil. Map of Brazil showing the geographical distribution of the species examples of each family that can be found in the country. Each color mentions one of the species/family and is marked in the state where it can be found. (**A**) *Liotyphlops ternetzii* (Anomalepididae), photo by Marco Vicariotto. (**B**) *Trilepida macrolepis* (Leptotyphlopidae), photo by Geovane Lima. (**C**) *Amerotyphlops reticulatus* (Typhlopidae), photo by Anthony Giardenelli. (**D**) *Anilius scytale* (Aniliidae), photo by Jonghyun Park. (**E**) *Tropidophis paucisquamis* (Tropidophiidae), photo by Janie Jones. (**F**) *Corallus hortulanus* (Boidae), photo by Guilherme Melo Dos Santos. (**G**) *Leptodeira ashmeadii* (Colubridae), photo by Guilherme Melo Dos Santos. (**H**) *Dipsas catesbyi* (Dipsadidae), photo by Guilherme Melo Dos Santos. (**I**) *Bothrops Atrox* (Viperidae), photo by Guilherme Melo Dos Santos. (**J**) *Micrurus hemprichii* (Elapidae), photo by Pedro Bisneto.

Currently, in Brazil, there are approximately 412 species of snakes with various habits, ranging from fossorial to aquatic, with the main families being *Anomalepididae*, *Leptotyphlopidae*, *Typhlopidae*, *Aniliidae*, *Tropidophiidae*, *Boidae*, *Colubridae*, and *Dipsadidae* for the non-venomous families and *Viperidae* and *Elapidae* for the venomous ones (Figure 2) [21,22].

The venomous snakes’ genera in Brazil encompass members of the *Viperidae* family (including the subfamily *Crotalinae*, generally called pit vipers), such as *Crotalus*, *Bothrops*, and *Lachesis*, as well as the *Elapidae* family, represented by the genus *Micrurus* (Figure 1B) [23]. According to the epidemiological bulletin issued by the Ministry of Health through the Secretariat of Health Surveillance and Environment in 2023, the northern (Acre, Rondônia, Roraima, Amazonas, Pará, Tocantins, and Amapá) and northeastern (Maranhão, Piauí, Bahia, Ceará, Rio Grande do Norte, Paraíba, Pernambuco, Alagoas, and Sergipe) regions of the country were classified among the five regions of the Brazilian territory that are more impacted by snakebite incidents (Figure 1C) [24]. Although the northern region of Brazil has a smaller population compared to other regions, the incidence of snakebites remains disproportionately high. Over 44% of all snakebites occur in the Amazon region, where the rate is five times higher than in any other state, despite the area accounting for only 8.7% of the country’s total population [25]. This underscores the urgent need to address snakebite prevention and treatment in these underserved and vulnerable areas.

### 2.1. Bothrops Snakes, a Widely Distributed and Medically Important Genus

*Bothrops* snakes are among the four genera of snakes with the greatest epidemiological relevance in Brazil. They are the leading cause of snakebites in the country, mirroring the trend observed in Central and South America [4,26]. In Brazil, species belonging to this genus are popularly known as *jararaca*, *jararacuçu*, *urutu-cruzeiro*, *comboia*, and *caiçaca* [26] and, worldwide, are also known as lancehead pit vipers [27]. There are 49 species of *Bothrops* snakes identified worldwide (Table 1 and Table 2). Interestingly, 31 species (~63%) are found in the Brazilian territory.

The high diversity of *Bothrops* snakes in Latin America leads to a considerable number of deaths and disabilities. *Bothrops*-related snakebites account for approximately 90% of envenoming cases across the country [29,30]. In Brazil, snakebites remain a significant public health issue [31,32], with the country being ranked as one of the three global capitals of snakebites [33]. Brazil encounters the third highest incidences of venomous snakebites after India and Sri Lanka [34].

From a more regional perspective, analyzing the cases in other South America countries, it occupies the first place. Snakes of the genus *Bothrops* are involved in 70% to 90% of reported cases, averaging 27,000 to 29,000 annual cases with a lethality rate of 0.5% [23,35,36,37]. This number is likely to be underestimated due to complex geographical barriers and socioeconomic factors.

The true impact of snakebite accidents is underestimated due to under-reporting in countries with developing economies such as Brazil, suggesting that the number of unrecorded cases is significantly higher than the number of reported ones [25,38]. Many factors can impact the notification of these snakebites, such as the difficulty of transporting the patients involved in bites in rural areas, under-reported deaths and cases in the official surveillance system (almost 30%), and the lack of post-envenomation medical follow-up, which complicates the reporting of deaths, even after the administration of antivenom. Furthermore, the availability and accessibility of antivenom are not uniform across the Brazilian territory, being concentrated in urban areas and leaving rural areas vulnerable, especially since 87% of the cases to date have occurred in rural regions [38]. In the Brazilian Amazon region, each municipality has only one hospital that provides the antivenom treatment. As there is no antivenom available in the rural health units near where most of the accidents occur, minorities such as riverine and Indigenous inhabitants are denied the possibility of timely and adequate treatment [25]. Moreover, these incidents are concentrated in areas with limited access to the treatment, leading to snakebite victims to start using traditional homemade medicines and inadequate prevention or first aid procedures (tourniquets, zootherapy, and phytotherapy), which contribute to the emergence of severe and under-reported cases and snakebite-related fatalities, also impacting the victim’s life quality [31,39].

### 2.2. Bothrops-Induced Clinical Manifestations

In general, bothropic envenoming can result in a complex pathology combining local and systemic effects induced by the action of tissue-damaging and hemotoxic toxins [40,41]. The severity varies depending on several factors but requires immediate attention due to acute emergencies and the fast action of venom proteins, which can lead to functional impairments or even death [42]. The clinical picture is well documented and used in hospital settings to determine the appropriate antivenom. Variations, such as previously observed chronic kidney failure and acute mesenteric ischemia [27,43], can be pointed out depending on the snake species and geographical locations, along with some rare clinical manifestations. These uncommon cases require further documentation and investigation to raise awareness and assist clinicians in making decisions and choosing appropriate and prompt interventions. In the following sections, we detail the common local and systemic events following *Bothrops* snakebites.

#### 2.2.1. Local Envenomation Effects

**Figure 3:**
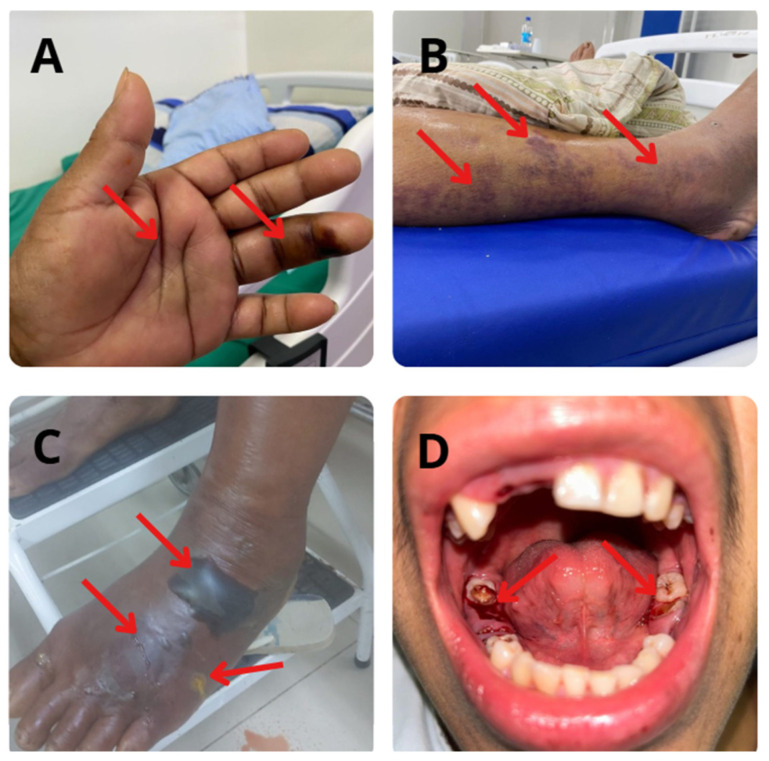
Local and systemic effects following bothropic envenomation. (**A**) Edema and onset of necrotizing process. (**B**) Edema and ecchymosis. (**C**) Edema and blisters. (**D**) Gum hemorrhage. All images are sourced from the image archive of the ITox-Lab research group. All patients provided written consent for image use during the research study.

The local effects of bothropic envenoming are widely recognized due to the extensive tissue damage and limited neutralizing properties of the antivenom at the bite site [44]. The main local clinical manifestations include edema, blister formation, epithelial damage, pain, and vascular alterations such as hemorrhage and blood incoagulability, as well as damage to skeletal muscle tissue, such as muscle degeneration and myonecrosis (Figure 3) [29]. Inflammation plays a crucial role, triggered by venom-derived toxins through three main mechanisms: the direct recognition of venom components by leukocyte receptors, indirect inflammatory response induced by damage-associated molecular patterns, and direct activation of complement system mediators by toxins [45].

These effects are the result of the multifactorial and synergistic actions of toxins, which are still poorly understood, acting on connective and muscular tissues and inducing the release of newly synthesized or stored endogenous inflammatory mediators [46,47]. Such responses trigger vascular and cellular events, characterized by vascular changes in the blood vessel caliber and flow, edema, cellular migration from the microcirculation, and accumulation at the injury site via chemotaxis and the activation of various inflammatory mediators [46].

Snake venom metalloproteinases (SVMPs) cause direct damage to microvessels, promoting increased permeability and extravasation, leading to vascular disturbances such as edema and hemorrhage [47,48,49,50]. SVMPs are also capable of activating specific inflammatory cells and mediators frequently associated with hyperalgesia [47,51]. Phospholipase A_2_ (PLA_2_) is involved in the enzymatic activity of membrane phospholipids, releasing eicosanoid precursors and contributing to inflammation-associated damage. This damage can progress to necrosis and, in many cases, lead to a loss of function or amputation of the affected limbs. Myonecrosis may occur due to the direct action of myotoxic PLA_2_ on the muscle cell membrane through changes induced by SVMPs or by other myotoxic proteins that disrupt the ionic control of muscle fibers. Another aspect of this process is the infiltration of leukocytes at the injury site, which release inflammatory mediators such as eicosanoids and cytokines, further enhancing the recruitment of phagocytic cells and contributing to necrosis [47].

#### 2.2.2. Systemic Effects

Bothropic envenoming can trigger severe systemic effects such as coagulopathy, thrombocytopenia, and severe hemorrhage, which can lead to death if not treated promptly [52]. Blood incoagulability is common, resulting in bleeding at the site of the bite, in the gums, and in vital organs (urinary, gastrointestinal, pulmonary, and central nervous systems) (Figure 3). Another common event includes thrombocytopenia, which can also increase the risk of bleeding. Systemic myotoxicity causes elevations in serum myoglobin and creatine kinase, which can lead to acute kidney injury, while the venom also induces oxidative stress, hepatotoxicity, and pulmonary changes such as respiratory failure and acute pulmonary edema, contributing to mortality [29,53,54]. Renal changes are concerning, with acute kidney injury (AKI) being a leading cause of mortality, and the pathogenesis of this condition is not yet fully understood, but it is known that venoms can trigger nephrotoxic and/or ischemic mechanisms in the body [54,55,56,57]. Studies with isolated kidneys have revealed varied impacts on renal function parameters depending on the venom composition [29,30]. Moreover, rare manifestations (e.g., mesenteric ischemia), long-term disabilities (e.g., permanent sequelae in gait and amputations), and psychological morbidity [7,8,43,58] have been also documented.

### 2.3. Biochemistry of Bothrops Venoms

**Figure 4:**
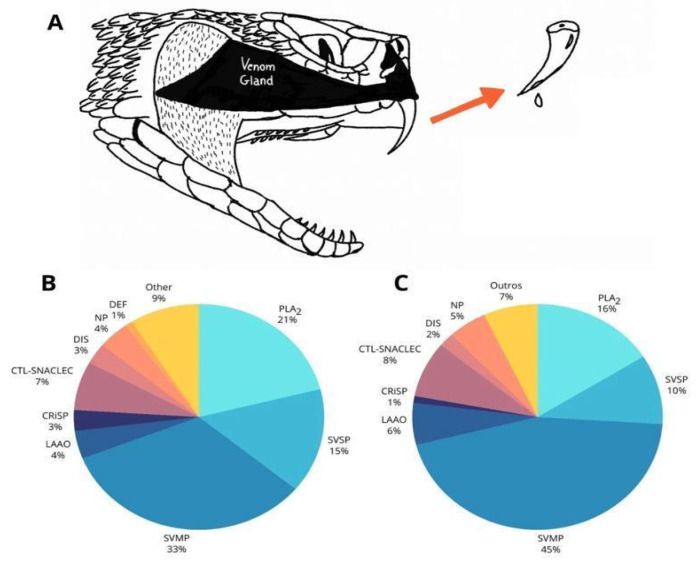
Production and biochemical composition of *Bothrops* venoms. (**A**) Anatomical arrangement of the snake venom gland and solenoglyphous dentition. (**B**) General composition of snake venom from the *Viperidae* family. (**C**) Biochemical trend the venom composition of *Bothrops* snakes. CRiSP (cysteine-rich secretory protein); CTL/SNACLEC (C-type lectin and C-type lectin-like protein); DEF (defensin); DIS (disintegrin); KSPI (Kunitz-type serine protease inhibitor); LAAO (L-amino acid oxidase); NP (natriuretic peptide); PLA_2_ (phospholipase A_2_); SVMP (snake venom metalloproteinase); SVSP (snake venom serine protease); and other minor venom components (unidentified components or components with an average abundance of <1%). Adapted from [7].

Similar to other snakes, *Bothrops* venoms are produced and secreted by modified salivary glands, primarily containing multifunctional protein-based toxins. These biomolecules and peptides are injected directly into the prey through specialized and highly efficient solenoglyphous dentition (Figure 4A) [36].

**Figure 5:**
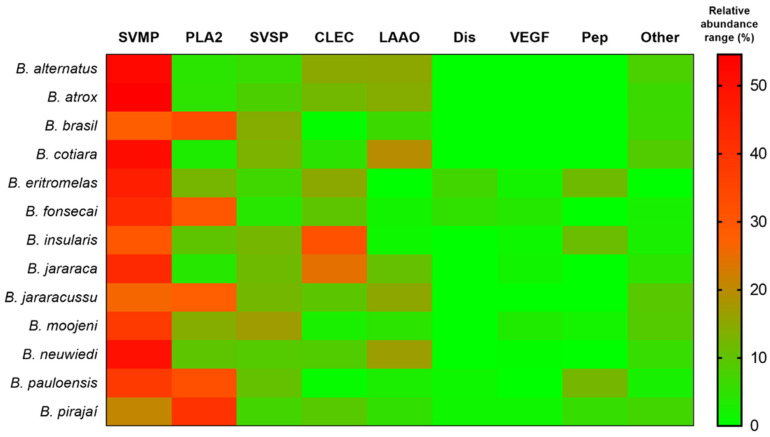
Heatmap of components identified in Brazilian bothropic venoms. SVMP (snake venom metalloprotease); PLA_2_ (phospholipase A_2_); SVSP (snake venom serine protease); CLEC (C-type lectin); LAAO (L-amino acid oxidase), Dis (disintegrin); VEGF (vascular endothelial growth factor); Pep (peptides); and other minor venom components (phosphodiesterase, CRISP, nerve growth factor, hyaluronidase, nucleotidase, peptidase, phospholipase inhibitor, glutaminyl cyclase, actin, and undetermined venom components). Adapted from [26].

Bothropic venoms are recognized for their biochemical complexity, with more than twenty catalogued toxins and over 90% of their dry weight consisting of protein material mainly represented by proteolytic enzymes (Figure 4B,C) [59,60,61]. Approximately 50% of *Bothrops* venoms are composed of SVSPs and SVMPs. For this reason, their main pathological effects derive from their proteolytic action, which induces a series of local and systemic complications [62]. This cocktail triggers inflammatory and coagulant events as well as necrotic and hemorrhagic activities [26,36]. The biochemistry of *Bothrops* venoms is dynamic, with intriguing variations that affect the action and neutralization capacity of antivenom [63,64]. The protein composition of *Bothrops* venom varies not only between species but also within the same species due to environmental factors, the age, sex, or type of prey available for feeding in the environment [7,8]. The diversity of elements present in this library impacts the wide range of effects caused by the venom, further affecting the production of antivenoms [65,66,67]. Although the venom of *Bothrops* snakes is remarkably varied, proteomic methods have enabled the meticulous identification of its main constituents [26]. Figure 5 summarizes the composition pattern of Brazilian bothropic venoms studied using mass spectrometry-based tools.

## 3. Introduction to Snake Venom Serine Proteases: From Catalysis to Functions

Serine proteases constitute 4–29% of *Bothrops* venoms. They are monomeric glycoproteins with a molecular mass between 25 and 70 kDa, exhibiting N or O-glycosylation sites [7,8,26,68,69]. The structure of SVSPs can also resemble chymotrypsin-like enzymes, in which the active site cleft is located at the junction of the two six-stranded beta barrels [70]. These proteases are structurally composed of a highly conserved catalytic site that includes the catalytic triad, formed by a reactive serine residue (Ser 195), which plays a role in the formation of a transient acyl-enzyme complex stabilized by the presence of histidine (His 57) and aspartic acid (Asp 102) residues within the active site. Moreover, SVSPs normally contain twelve cysteine residues that form six disulfide bridges and stabilize the structure of the proteins. One of these bridges (Cys91-Cys245) is unique and conserved only among SVSPs [7,8,26,70]. Despite most SVSPs sharing the classic reaction mechanism of serine proteases, over 20 SVSPs present variations in the canonical catalytic triad, as identified through venom transcriptomes [7,8].

Functionally, SVSPs constitute a group of blood coagulation factor-targeting enzymes, similar to thrombin. Considered hemotoxins, they are widely studied for their interaction with many components of the hemostatic system. In addition, they affect platelet aggregation, various aspects of coagulation cascades, fibrinolytic and kallikrein–kinin systems, and even other cells. Overall, this spectrum of activities causes an imbalance in the hemostatic system, leading to blood clotting disturbances following snakebite envenoming [7,8,26,70,71].

Many SVSPs are classified as thrombin-like enzymes (TLEs), which mainly affect blood coagulation cascades [72,73]. Intriguingly, they reproduce the effects of thrombin by presenting fibrinogenolytic activity similar to that of plasma thrombin. As a result, they convert fibrinogen to fibrin and activate factor V and protein C [7,8,26,68,71]. Some examples of TLEs from *Bothrops* snake venoms are batroxobin and TL-BJ [68,69]. Individually, SVSPs can exhibit one or more thrombin-like activities, but there are no records of an SVSP fully resembling thrombin and possessing all of its bioactivities. Furthermore, SVSPs may exhibit bioactivities that are not expressed by thrombin. This difference between SVSPs and thrombin makes SVSPs responsible for disrupting homeostasis [7,8].

Unlike thrombin, SVSPs are highly specific for macromolecular substrates, activating or inhibiting various coagulation factors such as factor V, factor VIII, and fibrinogen [1,74,75]. Due to such specificity exhibited by SVSPs, a group of isoforms can induce different physiological manifestations due to the high degree of mutual identity, ranging from 50 to 85%, known as the identity-selectivity paradox, in which specificity cannot be understood from a primary sequence of SVSPs. This is a puzzle that deserves further investigation. The precise specificity and intense hemotoxicity of SVSPs have led to their study as potential diagnostic and therapeutic tools for coagulopathies and heart diseases [7,8,26,70].

Some studies have demonstrated the ability of SVSPs to release bradykinin (BK) or kallidin (Lys-BK) through the hydrolysis of kininogen, such as crotalase, elegaxobin II, and KN-BJ SVSPs, which are known as kallikrein-like enzymes [68]. Bradykinin, an anti-inflammatory plasma peptide that is active in vascular smooth muscle, resulting in vasodilation and increased vascular permeability, is capable of reducing blood pressure when released from kininogen [76,77]. Thus, these kallikrein-like enzymes are molecules that contribute to the evolution of hypotensive shock in victims of snakebite envenomation [68,69,71].

## 4. *Bothrops* Venoms: A Rich Source of Serine Proteases

Bothropic venom contains higher levels of serine proteases compared to other snake venoms. The data retrieved from UniProt indicate 43 serine proteases from crotalid venoms, 8 from lachetic venoms, and no results for serine proteases derived from *Micrurus* spp. venoms. Interestingly, the number of serine proteases found in Bothropic snakes totals 48, according to database searches (Table 3). The serine proteases derived from the venom of Brazilian *Bothrops* snakes are highlighted in green, while other serine proteases from *Bothrops* snake venoms from different countries are marked in blue. Overall, the literature and database search pointed out that Brazilian snake venoms have significantly contributed to this field of study, with the highest number of entries (88%).

## 5. Unveiling the Enzymatic Nature of Serine Proteinases

Serine proteinases belong to the trypsin family S1 of clan SA, the largest family of peptidases [89]. Despite the high degree of sequence identity among SVSPs, they exhibit a remarkable specificity toward macromolecular substrates, with highly conserved S1 subsites, and high selectivity towards macromolecular substrates, such as blood coagulation factors S-2222 (plasmin and trypsin), S-2238 (thrombin), S-2266 (plasmin), S-2251 (plasmin), and S-2288 (plasmatic kallikrein and thrombin). In all of these, the product p-nitroanilin (pNA) is a compound that, after cleavage, can be easily detected via spectrophotometry at 405 nm [90,91]. Bothrombin from *Bothrops jararaca* mimics thrombin by cleaving fibrinogen but does not induce clot formation, while Ancrod from *Calloselasma rhodostoma* depletes fibrinogen and is used therapeutically for anticoagulation. Thrombin-like enzymes (TLEs) from *Bothrops atrox* selectively cleave fibrinogen without causing full clotting. On the other hand, RVV-X from *Daboia russelii* activates factor X, and PA-BJ from *Bothrops jararaca* acts as a plasminogen activator, aiding in fibrinolysis. These enzymes demonstrate the broad range of SVSPs in modulating blood processes [92,93,94,95,96].

Both acidic and basic serine proteinases have been isolated from snake venoms, with their isoelectric point (pI) varying from 3.5 to 6.2. Basic serine proteinases, often in the range of 8.0 to 9.5, typically exhibit direct platelet-aggregating activity, whereas acidic serine proteinases, usually falling between 4.5 and 6.0, are associated with a range of proteolytic activities on hemostasis-related substrates [97]. Different SVSP preparations exhibit distinct forms that can be observed on electrophoresis, with slightly varying molecular weight and pIs, resulting from the differences in their amino acid sequences and glycosylation levels [98].

**Figure 6:**
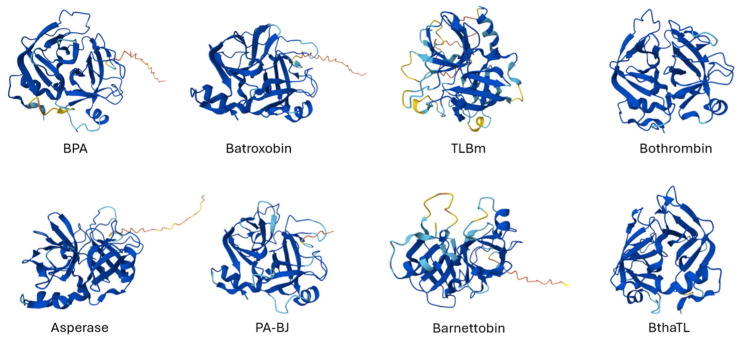
AlphaFold-predicted structures of serine proteases. Three-dimensional structures of serine proteases from different *Bothrops* species predicted by AlphaFold. The sequences for Asperase and Barnettobin remain incomplete, while the other serine proteases shown were fully sequenced. The corresponding three-dimensional structures for additional serine proteases can be accessed on UniProt using the identifier numbers listed in Table 3. Adapted from UniProt.

Structural analysis (Figure 6) of SVSPs has suggested the importance of key specific elements that might be responsible for their high substrate selectivity [99,100,101]. The catalytic triad (His57, Asp102, and Ser195) is positioned at the junction between the two barrels and is surrounded by the conserved 70, 148, and 218 loops, as well as the non-conserved 37, 60, 99, and 174 loops. Catalytic residue His57 possesses a non-optimal Nd1-H tautomeric conformation, which is essential for catalysis [99,100]. The catalytic triad is supported by an extensive hydrogen bonding network formed between the Nd1-H of His57 and Od1 of Asp102, as well as between the OH of Ser195 and the Ne2-H of His57. For this, they are all sensitive to the serine-modifying reagents phenylmethylsulphonyl fluoride (PMSF) and diisopropylfluorophosphate (DFP) [97].

The isolation from snake venoms can be followed by the assessment of the activity on chromogenic or fluorogenic peptide substrates containing arginine or lysine at the P1 position, which can measure the amidolytic activity of SVSPs. These molecules are useful to measure the amidolytic activity of SVSPs [102,103,104]. The hydrolytic activity of SVSPs on these peptide substrates is heavily influenced by the peptide moiety adjacent to the scissile bond. Like trypsin, the S1 subsite and oxyanion hole are only formed upon cleavage and removal of the peptide, as the N-terminal portion is conserved in both snake and mammalian enzymes [105]. Consequently, as with other serine proteinases, the loss of protease activity at a high pH is likely due to the deprotonation of the N-terminus and the disruption of the salt bridge. This disruption shifts the conformational equilibrium towards an inactive, zymogen-like state [106]. According to our literature search, studies have employed natural and synthetic substrates to study the catalytic properties of serine proteases, including kinetic parameters. The most common synthetic protein-based substrates are Nα-benzoyl-l-arginine 7-amido-4-methyl coumarin HCl and α-benzoyl-DL-arginine 4-nitroanilide hydrochloride [107]. Another alternative is the use of azocasein in an absorbance-based assay [108]. However, this is a general protein-mimicking substrate that can also detect SVMPs. Therefore, it must be used in presence of an SVMP inhibitor, such as EDTA. Finally, some researchers have worked with natural substrates, such as fibrinogen, via an electrophoresis-based approach.

## 6. Serine Proteases: From Toxic Effects to Drug Templates and Clinical Use

**Figure 7:**
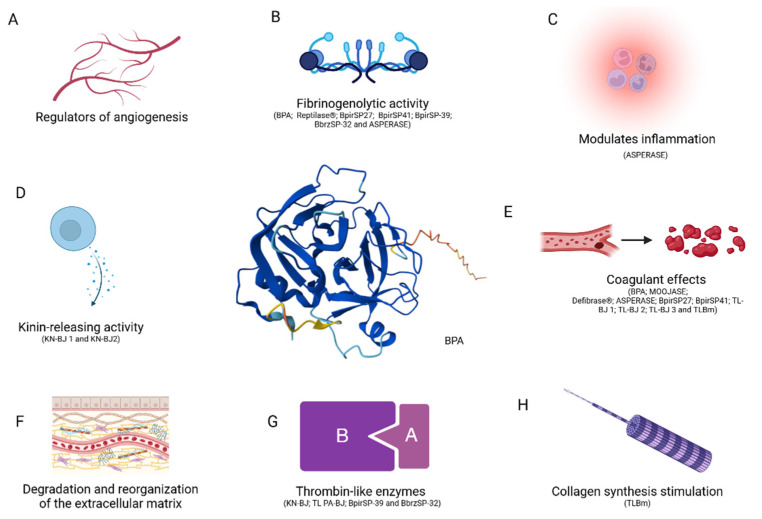
Spectrum of biological activities of serine proteases and the serine protease substrates and drugs derived from bothropic venom. In the middle of the figure, we represent a serine protease with the tridimensional AlphaFold structure of BPA isolated from *Bothrops jararaca*. (**A**) Serine proteases release and modify growth factors in angiogenesis (e.g., chymotrypsin, trypsin, elastase, and plasmin). (**B**) Fibrinogenolytic activity correlated with degradation and human fibrinogen cleavage (α and β chains) and fibrin production (e.g., BPA, BpirSP27, BpirSP41, BpirSP-39, BpirSP-32, and Asperase as serine proteases and Reptilase^®^ as a drug derived from an SVTLE isolated from *Bothrops atrox* venom). (**C**) Reduces the production of inflammatory mediators and reduce cell migration (e.g., Asperase). (**D**) Kinin-releasing activity (e.g., KN-BJ 1 and KN-BJ 2). (**E**) Antithrombotic effects and clotting degradation (e.g., BPA, Moojase, Asperase, BpirSP27, BpirSP41, TL-BJ 1, TL-BJ 2, TL-BJ 3, and TlBm and Defibrase^®^ as a drug derived from serine proteases). (**F**) Degradation and reorganization of the extracellular matrix (e.g., chymotrypsin, trypsin, elastase, and plasmin). (**G**) Thrombin-like enzymes (e.g., KN-BJ, PA-BJ, BpirSP-39, and BpirSP-32). (**H**) Collagen synthesis stimulation (e.g., TLBm).

There is a growing movement of studies involving serine proteases from *Bothrops* ssp. venoms due to their diverse applications (Figure 7), ranging from modulating blood coagulation, fibrinolysis, and platelet aggregation activation to regulating the inflammatory response [87]. This wide range of therapeutic applications highlights the significant potential of *Bothrops* ssp. serine proteases in modern medicine [81].

Serine proteinases (SVSPs), including chymotrypsin, trypsin, elastase, and plasmin, play a crucial role as regulators of angiogenesis [109,110]. These enzymes are activated through the cleavage of their N-terminal pro-peptide domains, resulting in the exposure of their catalytic triad; consequently, this process further stimulates the degradation and reorganization of the extracellular matrix, promoting cell migration and invasion. Additionally, these enzymes play an important role in releasing and modifying growth factors that drive the angiogenesis process in cancer patients [111].

Different *Bothrops* species have been categorized, along with their corresponding serine proteases. The *Bothrops jararaca* species, for instance, has numerous serine proteases described with their corresponding actions. The most glycosylated SVSP, *Bothrops* A protease (BPA), is found in bothropic venom, exhibiting potent in vitro fibrinogenolytic action with the ability to cleave human fibrinogen at an extremely low enzymatic concentration [96,112]. Due to its efficacy and thermostability, BPA can be applied in preventing thrombus formation. Three other serine proteases from jararaca, TL-BJ, KN-BJ, and PA-BJ, present distinct activities and isoforms [78,113,114]. TL-BJ exhibits coagulant activity similar to that of thrombin, with three different electrophoretic forms: TL-BJ 1, TL-BJ 2, and TL-BJ 3. Still presenting coagulant action, another serine called KN-BJ exhibits kinin-releasing action, with two distinct molecular weight forms: KN-BJ 1 and KN-BJ 2. PA-BJ shows a greater degree of similarity to thrombin-like enzymes from snake venom, trypsin, and tissue kallikrein than to thrombin itself [115]. Besides presenting different isoforms, studies have revealed that different cDNAs have open-reading regions, which when transcribed, can lead to the same amino acid sequence. Regarding this characteristic, three positive cDNAs can be mentioned—HS 112, HS 114, and HS 120—which lead to similar amino acid sequences present in serine KN-BJ 2 [78,116]. Each new study draws attention due to the abundance and richness observed in the venoms [78].

Batroxobin (Reptilase^®^) is a drug derived from an SVTLE isolated from *Bothrops atrox* venom, demonstrating the ability to cleave the α chain of fibrinogen, resulting in its conversion into loose fibrin monomers [85]. This drug exhibits promising therapeutic potential in treating ischemia in animal rat models, as well as in vivo clinical applications [85]. Additionally, it has been employed in fibrin production, a biomaterial used as a surgical adhesive in procedures involving tissue traumas. A notable example is the drug Defibrase^®^, clinically used in patients suffering from thrombosis, myocardial infarction, peripheral vascular diseases, acute ischemia, and renal transplant rejection, acting as a defibrinogenating agent [117].

The isolated serine protease from *Bothrops moojeni*, named moojase, has been identified as a potential anticoagulant agent [5,118]. Its action involves cleaving the α and β chains of fibrinogen, resulting in the formation of an unstable clot. This serine protease has demonstrated resistance to thrombin inhibitors and acts specifically on fibrinogen without affecting platelet function [85,119].

Serine proteases (SVSPs) do not possess intrinsic lethality, and their toxicity is mainly related to their effects on the hemostatic system. Many SVSPs are classified as thrombin-like enzymes due to their ability to degrade fibrinogen and promote blood coagulation. In most cases, these enzymes specifically act on the Aα chain of fibrinogen, although some may preferentially act on the Bβ chain or both chains [75,120]. The specific cleavage of fibrinogen by *Bothrops pirajai* serine proteases is related to their ability to induce coagulation of human plasma [81,117]. Results from Dose-Dependent Concentration Measurement (DDCM) and coagulation kinetics have shown that BpirSP41 exhibits greater coagulant potential than BpirSP27, possibly due to their different actions on fibrinogen, with BpirSP27 degrading only the Bβ chain, while BpirSP41 acts on both the Aα and Bβ chains. In addition to their action on fibrinogen, it is likely that BpirSP27 and BpirSP41 promote plasma coagulation through interaction with other factors that participate in the coagulation cascade [117].

Additionally, two other snake venom serine proteases (SVSP), named BpirSP-39, isolated from *Bothrops pirajai*, and BbrzSP-32, isolated from *Bothrops brazili*, have shown interesting and promising results as alternatives to thrombin [81,121,122]. These proteases mimic various characteristics of thrombin. For instance, BpirSP-39 showed positive activity for factor XIII activation, the degradation of Aα and Bβ chains of fibrinogen, the degradation of the BAPNA substrate, and inhibition by PMSF [81,119]. Meanwhile, BbrzSP-32 demonstrated the ability to aggregate and disaggregate platelets, clot retraction, the degradation of the α chain of fibrinogen, the degradation of the BAPNA substrate, and inhibition by PMSF [119].

Asperase, originating from *Bothrops asper*, has been the subject of study due to its potential therapeutic applications in various medical conditions. It exhibits anticoagulant activity by inhibiting blood coagulation through the degradation of factors such as fibrinogen and other elements of the coagulation cascade [123]. Additionally, it demonstrates potential antithrombotic effects, preventing thrombus formation by interfering with platelet aggregation and coagulation processes [124]. It also exhibits anti-inflammatory properties, modulating the inflammatory response by reducing the production of inflammatory mediators and the migration of inflammatory cells to the site of injury [125].

The serine protease TLBm, derived from *Bothrops marajoensis*, despite its activity in the coagulation cascade, acting as an anticoagulant, antithrombotic, and fibrinolytic agent, also demonstrates potential as a wound healing agent that stimulates collagen synthesis, favoring tissue regeneration [126].

The translation of toxin-based drugs to the pharmaceutical market requires substantial investments in long-term programs that support all challenging phases of development, from the discovery to clinical trials. As exemplified in this study, most of the applications have been identified in laboratory settings using in vitro techniques. However, successful examples that have reached the market and advanced stages of development were also provided, illustrating the applicability of serine proteases. Modern techniques and the adventure of artificial intelligence can drive innovation and facilitate the discovery and translation of serine proteases. This field has the potential to address important global issues, involving cardiovascular diseases and snakebites.

## 7. Concluding Remarks

Serine proteases derived from *Bothrops* venoms exhibit multiple chemical structures and variations, providing a broad spectrum of therapeutic applications. Due to their unique properties and significant influence on the coagulation cascade, these enzymes have been widely studied for their remarkable anticoagulant, antithrombotic, fibrinolytic, and even healing activities, potentially applied in promoting tissue regeneration. Advances in research on their characteristics and underlying mechanisms of action have substantially contributed to our current understanding of the pathophysiology associated with coagulation disorders and snakebites. This review has also fueled the emergence of new promising perspectives in the development of drugs and innovative therapies.

## Figures and Tables

**Table 1:** *Bothrops* species have a wide range of distribution in Brazil.

Snake	Geographical Distribution	Region of Brazil	States of Brazil
*B. alcatraz*	Brazil	Southeast	SP
*B. alternatus*	Argentina, Brazil, and Uruguay	South, Southeast, And Midwest	MS, GO, MG, RJ, SP, PR, SC, and RS
*B. atrox*	Bolivia, Brazil, Colombia, Ecuador, English Guiana, French Guiana, Peru, Suriname, Trinidad, and Venezuela	North, Midwest, and Northeast	RR, AP, AC, RO, AM, PA, TO, MT, and MA
*B. bilineatus*	Bolivia, Brazil, Colombia, Ecuador, English Guiana, French Guiana, and Suriname	North, Northeast, Southeast, and Midwest	RR, AP, RO, AM, PA, MT, CE, PE, AL, BA, ES, MG, and RJ
*B. brazili*	Bolivia, Brazil, Colombia, English Guiana, French Guiana, Peru, Venezuela, and Suriname	North, Northeast, and Midwest	AC, RO, AM, PA, MT, and MA
*B. cotiara*	Argentina and Brazil	Southeast and South	SP, PR, SC, and RS
*B. diporus*	Argentina, Bolivia, Brazil, and Paraguay	Southeast and South	SP, PR, SC, and RS
*B. erythromelas*	Brazil	Northeast and Southeast	PI, CE, RN, PB, PE, AL, SE, and BA
*B. fonsecai*	Brazil	na	MG, RJ, and SP
*B. germanoi*	Brazil	Southeast	SP
*B. insularis*	Brazil	Southeast	SP
*B. itapetiningae*	Brazil	Midwest, Southeast, and South	MS, GO, DF, MG, SP, and PR
*B. jabrensis*	Brazil	Northeast	PB
*B. jararaca*	Argentina, Brazil, and Paraguay	Midwest, Southeast, and South	GO, BA, ES, MG, RJ, SP, PR, SC, and RS
*B. jararacussu*	Argentina, Bolivia, Brazil, and Paraguay	Northeast, Southeast, and South	BA, ES, MG, RJ, SP, PR, SC, RS, and MS
*B. leucurus*	Brazil	Northeast and Southeast	CE, RN, PB, PE, AL, SE, BA, ES, and MG
*B. lutzi*	Brazil	North, Midwest, and Southeast	TO, GO, DF, MA, PI, CE, PE, BA, and MG
*B. marajoensis*	Brazil	North and Northeast	AP, PA, and MA
*B. marmoratus*	Brazil	North, Midwest, and Southeast	TO, GO, DF, and MG
*B. mattogrossensis*	Bolivia and Brazil	North, Midwest, and Southeast	RO, AM, TO, MT, MS, GO, and SP
*B. moojeni*	Argentina, Bolivia, Brazil, and Paraguay	North, Midwest, Southeast, and Northeast	TO, MT, MS, GO, DF, MA, PI, BA, MG, SP, and PR
*B. muriciensis*	Brazil	Northeast	AL
*B. neuwiedi*	Argentina and Brazil	Midwest, Southeast, and South	GO, DF, BA, MG, RJ, SP PR, SC, and RS
*B. oligobalius*	Bolivia, Brazil, Colombia, English Guiana, French Guiana, Peru, Venezuela, and Suriname	North	AP, AM, and PA
*B. otavioi*	Brazil	Southeast	SP
*B. pauloensis*	Bolivia and Brazil	Midwest and Southeast	MT, MS, GO, DF, MG, SP, and PR
*B. pirajai*	Brazil	Northeast	BA
*B. pubescens*	Brazil and Uruguay	South	SC and RS
*B. sazimai*	Brazil	Southeast	ES
*B. smaragdinus*	Bolivia, Brazil, Colombia, Ecuador, English Guiana, French Guiana, and Suriname	North	AC, RO, and AM
*B. taeniatus*	Bolivia, Brazil, Colombia, Ecuador, English Guiana, French Guiana, and Peru	North, Midwest, and Northeast	RR, AP, AC, RO, AM, PA, MT, and MA

RR—Roraima, AP—Amapá, AC—Acre, RO—Rondônia, AM—Amazonas, PA—Pará, TO—Tocantins, MT—Mato Grosso, MS—Mato Grosso do Sul, GO—Goiás, DF—Distrito Federal, MA—Maranhão, PI—Piauí, CE—Ceará, RN—Rio Grande do Norte, PB—Paraíba, PE—Pernambuco, AL—Alagoas, SE—Sergipe, BA—Bahia, ES—Espirito Santo, MG—Minas Gerais, RJ—Rio de Janeiro, SP—São Paulo, PR—Paraná, SC—Santa Catarina, and RS—Rio Grande do Sul. Adapted from [19,26,28].

**Table 2:** *Bothrops* species distribution in South and Central America, excluding Brazil.

Snake	Geographical Distribution
*B. ammodytoides*	Argentina
*B. asper*	Belize, Colombia, Costa Rica, Ecuador, Guatemala, Honduras, Nicaragua, Mexico, Panama, Peru, and Venezuela
*B. ayerbei*	Colombia
*B. barnetti*	Peru
*B. caribbaeus*	Saint Lucia and Antilles
*B. chloromelas*	Peru
*B. jonathani*	Argentina and Bolivia
*B. lanceolatus*	Antilles
*B. medusa*	Venezuela
*B. monsignifer*	Bolivia
*B. oligolepis*	Peru
*B. osbornei*	Ecuador and Peru
*B. pictus*	Peru
*B. pulcher*	Colombia and Ecuador
*B. punctatus*	Colombia, Ecuador, and Panama
*B. sanctaecrucis*	Bolivia
*B. sonene*	Peru
*B. venezuelensis*	Venezuela

*Bothrops* snake species that can be found in South and Central America, excluding Brazil as one of the countries. All other species that can also be found in Brazil and other South and Central American countries can be found in Table 1. Adapted from [19,26,28].

**Table 3:** *Bothrops*-derived serine proteases. Brazilian snake venoms have been an important source for enhancing our understanding of the structure and actions of these proteolytic enzymes.

Serine Protease	Snake	Geographical Location	Activities	Length (aa)	Molecular Weight (Da)	UniProt Number/Ref
BPA	*Bothrops jararaca*	Brazil (SP, RJ, BA, RS, SC, MG, GO, MT, ES, and PR)	Fibrinogenolytic, stereolytic, and amidolytic activities	258	~28.38	UniProt Q9PTU8
HS112			It acts on the hemostasis system of the prey	255	~28.05	UniProt Q5W960
HS114			Nonspecific action on fibrinogen; low fibrinolytic activity; high enzymatic activity when compared to plasmin	259	~28.49	UniProt Q5W959
SVSP like-HS120			*unknown*	253	28	[78,79]
Bothrombin			Similar to thrombin; induces platelet aggregation; activates factor VIII	232	~25.52	UniProt P81661
PA-BJ			Induces platelet aggregation; thrombin-like activity; amidolytic activity	237	~26.07	UniProt P81824
TL-BJ1			Similar to thrombin; causes specific coagulation of fibrinogen (FGA) with the release of fibrinopeptide A	19 *	* not reported *	UniProt P81882
TL-BJ2			Similar to thrombin; causes specific coagulation of fibrinogen (FGA) with the release of fibrinopeptide A	19 *	* not reported *	UniProt P81883
TL-BJ3			Similar to thrombin; causes specific coagulation of fibrinogen (FGA) with the release of fibrinopeptide A	19 *	* not reported *	UniProt P81884
KN-BJ1			Kinin releasing; activity depends on kininogen source; coagulation activities; clotting and fibrin formation	19 *	38	[80]
KN-BJ2			Kinin releasing; activity depends on kininogen source; coagulation activities; clotting and fibrin formation	19 *	39	[80]
Bhalternina	*Bothrops alternatus*	Argentina, Uruguay, and Brazil (RS, SP, RJ, MS, GO, MG, PR, and SC)	Similar to thrombin that induces blood coagulation coagulation in vitro; in vivo defibrinogenation; albuminolytic and fibrinogenolytic activities	260	~28.6	UniProt P0CG03
Balterina			* unknown *	* not reported *	* not reported *	(VILCA QUISPE, 2013)
BthatL			It acts on the hemostasis system of the prey	233	~25.63	UniProt Q6IWF1
Barnettobina	*Bothrops barnetti*	Peru	Similar to thrombin; releases only fibrinopeptide A from the human alpha chain of fibrinogen; fibrinogenolytic and defibrinogenating activities	249	39	UniProt K4LLQ2
BpirSP41	*Bothrops pirajai*	Brazil (BA)	Similar to thrombin; releases only fibrinopeptide A from the human alpha chain of fibrinogen; fibrinogenolytic and defibrinogenating activities	50 * (~364)	40	UniProt P0DL27
BpirSP-39			Coagulation activity; activation of factors XIIIa and III of the coagulation cascade; gelatinolytic activity; amidolytic activity	~445	49	[81]
BpirSP27			Preferably degrades the beta chain (FGB) of fibrinogen; promotes concentration-dependent platelet aggregation, in the presence or absence of calcium; hydrolyzes chromogenic substrates	50 * (~245)	27	UniProt P0DL26
Asperase	*Bothrops asper*	Mexico, Guatemala, Honduras, Nicaragua, Costa Rica, Panama, Belize, Colombia, Ecuador, and Venezuela	Defibrillation; coagulation of human plasma and bovine fibrinogen; when administered intravenously induces effect similar to that of gyroxin	259	27	UniProt Q072L6
TLBm	*Bothrops marajoensis*	Brazil (AP and PA)	Similar to thrombin; induces platelet aggregation	285	33	UniProt P0DJE9
Batroxobina	*Bothrops atrox*	Brazil (RR, AM, AP, AC, PA, MA, RO, TO, and MT)	Similar to thrombin; cleaves Arg-Gly ligations in fibrinogen alpha chains (FGAs)	255	28	UniProt P04971
Ba III-4			Similar to thrombin; coagulant activity; fibrinogenolytic activity; proteolytic activity; alkaline phosphatase	293	34	[82]
Thrombocytin			Thrombin-like α-chain; fibrinogenolytic; platelet activator	~327	36	[83]
Leuurobina	*Bothrops leucurus*	Brazil (BA, CE, RN, PB, PE, AL, SE, ES, and MG)	Similar to thrombin; induces temporary opisthotonos episodes; intravenous administration produces an effect similar to that of gyroxine	231	30	UniProt P0DJ86
Leucurobin			Thrombin-like α-chain; fibrinogenolytic	~318	35	[83]
BJ-48	*Bothrops jararacussu*	Brazil (MS, BA, ES, MG, RJ, SP, PR, SC, and RS)	Similar to thrombin; specifically cleaves the alpha chain of human fibrinogen (FGA); selective for Arg over Lys at position 1 of the tripeptide substrate	22 * (~436)	48	UniProt P0DJF0
DV			Similar to thrombin; induces the rapid formation of fibrin clots	10 *	* not reported *	[5]
BjussusSP-1			Similar to thrombin; fibrinogenolytic activity specific to alpha chain (FGA); hydrolyzes fibrin, BAPNA, TAME, and artificial chromogenic substrates of the coagulation cascade	232	~25.52	UniProt Q2PQJ3
FC-Bj			Thrombin-like α/β-chain; fibrinogenolytic	* not reported *	* not reported *	[83]
Jararacussin-I			Thrombin-like α/β-chain; fibrinogenolytic	~254	28	[83]
BJV-VIIIcp			Platelet activator	~254	28	[83]
Moojase	*Bothrops moojeni*	Brazil (TO, MT, MS, GO, DF, MA, PI, BA, MG, SP, and PR)	Similar to thrombin; fibrinogenolytic activity; fibrinolytic activity; high cleavage effectiveness for chromogenic substrates	50 *	36	[5]
				(~327)		
BMII32			Plasma coagulant activity; fibrinogenolytic action without inducing fibrinolysis	~290	32	[5]
BMII35			Plasma coagulant activity; fibrinogenolytic action without inducing fibrinolysis	~318	35	[5]
BmooSP			Coagulant activity; defibrinating activity; caseinolytic activity; fibrinogenolytic activity	~327	36	[5]
MSP 1			Platelet activator	~309	34	[83]
TI-Bp	*Bothrops pauloensis*	Paraguay, Bolivia, and Brazil (MT, MS, GO, DF, MG, SP, and PR)	* unknown *	* not reported *	* not reported *	[84]
rBpSP-I			Similar to thrombin; high coagulation activity in bovine and human plasma; high fibrinogenolytic activity on substrates such as TAME and specific substrates for thrombin; hydrolyzes substrates for kallikrein; intraperitoneal administration causes defibrinogenation	15 *	* not reported *	UniProt P0DJF1
rBpSP-II			* unknown *	~409	45	[85]
rBamSP_1	*Bothrops ammodytoides*	Argentina	* unknown *	~245	27	[86]
SVSP	*Bothrops fonsecai*	Brazil (MG, RJ, and SP)	It acts on the hemostasis system of the prey	15 *	* not reported *	UniProt P0DMH6
BITS01A	*Bothrops insularis*	Brazil (SP)	It acts on the hemostasis system of the prey	257	~28.27	UniProt Q8QG86
Pictobin	*Bothrops pictus*	Peru	It acts on the hemostasis system of the prey	250	~27.5	UniProt U5YCR8
TLBro			Coagulant activity; catalytic activity; proteolytic activity; fibrinogenolytic activity; fibrinolytic activity	183	20	[84]
SVSP	*Bothrops cotiara*	Argentina and Brazil (SP, PR, SC, and RS)	It acts on the hemostasis system of the prey	15 *	* not reported *	UniProt P0DMH5
TLBbz	*Bothrops brazili*	Venezuela, Guyana, Suriname, French Guiana, Colombia, Peru, Ecuador, Bolivia, and Brazil (PR, AM, RO, and MT)	Coagulant activity; catalytic activity; proteolytic activity; fibrinogenolytic activity; fibrinolytic activity	321	35	[84]
BbrzSP32			Proteolytic activity; thrombolytic activity; procoagulant activity; hydrolytic activity	~290	32	[87]
TL-Ban	*Bothrocopias andianus*	Peru and Bolivia	Coagulant activity; fibrinogenolytic activity; proteolytic activity	269	25	[88]

In green: serine proteases from *Bothrops* snakes found in Brazil; in blue: serine proteases from *Bothrops* snakes from other locations around the world. (*): fragment of serine proteases’ length (aa) regarding non-terminal residue; (~): estimated values (1 aa equivalent to 110 Da); (*unknown*): unknown or no described serine protease activity; (*not reported*): values not found in databases.

## Data Availability

Not applicable.
